# Effect of CYP2C19 polymorphisms on serum valproic level acid in Chinese Han patients with schizophrenia

**DOI:** 10.1038/s41598-021-02628-x

**Published:** 2021-11-30

**Authors:** S. Wang, J. Li, M. Song, P. Yan, X. Ju, J. Liu, C. Wang, J. Shi

**Affiliations:** 1 The Molecular Biology Laboratory, Hangzhou Seventh People’s Hospital, Hangzhou, 310013 China; 2 The Department of Psychiatric, Hangzhou Seventh People’s Hospital, Hangzhou, 310013 China; 3 Mental Health Center, Zhejiang University School of Medicine, Hangzhou, 310013 China

**Keywords:** Diseases of the nervous system, Genetics of the nervous system

## Abstract

Valproic acid is an anticonvulsant, which is also widely used for treating psychiatric disorders. Some clinical trials have demonstrated benefits of valproic acid augmentation therapy in schizophrenia. Interindividual variability in valproic acid dose and serum concentration may reflect functional consequences of genetic polymorphisms in genes encoding drug-metabolizing enzymes. The aim of this study was to determine the relationship between serum concentrations of valproic acid and single nucleotide polymorphisms of the *cytochrome P450 (CYP) 2C19* gene in patients with schizophrenia. All patients had been receiving fixed dose of valproic acid for at least 2 weeks. The daily doses were 0.5–1.5 g. No other drugs except olanzapine were coadministered. Serum concentrations of valproic acid were measured using the ultra-high performance liquid chromatography method with mass-spectrometric detection. The *CYP2C19 *(CYP2C19*2 G681A rs4244285 and CYP2C19*3 G636A rs4986893) genotypes were identified by real-time PCR analyses. The mean concentration/dose ratios of valproic acid were significantly higher in patients with CYP2C19 *1/*2 genotype (*P* < 0.01) or CYP2C19 *2/*3 genotype (*P* < 0.01) than in those with CYP2C12 *1/*1 genotype. The mean concentration/dose ratios of valproic acid were significantly higher in patients with 1 (*P* < 0.01) or 2 (*P* < 0.01) mutated alleles for *CYP2C19* than in those without mutated alleles. And the post hoc analysis revealed that the result has acceptable statistical (power (1 – β) = 0.8486 at type I  level of 0.05) to support the observed significant associations for *CYP2C19* SNPs and serum C/D ratios of valproic acid. The findings of this study suggest that the genetic polymorphisms of CYP2C19 significantly affect the steady-state serum concentrations of valproic acid in Chinese Han population. The determination of the CYP2C19 genotypes may be useful for dosing adjustment in schizophrenia patients on valproic acid therapy.

## Introduction

Over the last 40 years, a variety of adjunctive treatments have been used to treat schizophrenia^1^. These are often used in addition to antipsychotics, in an attempt to alleviate the symptoms of schizophrenia such as hallucinations and delusional beliefs, although they have been used instead of antipsychotics. Valproate/valproic acid has been used for people whose psychosis did not respond to traditional therapy. Valproic acid is traditionally used as an anticonvulsant drug and is also used for affective disorders, especially for the treatment of acute mania^2^. Furthermore, it is thought to have anti-aggressive effects and it may reduce impulsive behaviour, which might be useful for some people with schizophrenia^3–5^. The dose of valproic acid and the plasma concentration may be significantly different among individuals due to genetic polymorphisms of cytochrome P450 (CYP) 2C19 and 2C9, or interactions with other drugs^6,7^. Optimizing the valproic acid dose with genetic variation could play a key role in individualized care to avoid adverse drug reactions or treatment failure and maximize drug efficacy^8^.

The metabolism of valproic acid is complex. The major metabolic pathways of valproic acid comprise glucuronidation and mitochondrial β-oxidation, while CYP-mediated oxidation is only a minor pathway^9^. CYP2C19 plays a key role in the biotransformation of valproic acid in humans. There are significant racial differences in valproic acid transformation due to gene polymorphism^10^.

There is large interindividual variability in its pharmacokinetics and pharmacodynamics^7,11^. Therefore, its serum concentration needs to be monitored as a guide of dose adjustment during the course of therapy. Genetic factors have been reported to represent key factors that influence the pharmacokinetics variability of VPA. In particular, certain polymorphisms of cytochrome P450 2C19 (CYP2C19) has been shown to affect VPA pharmacokinetics^12,13^. Several mutated alleles of the *CYP2C19* locus that cause decreased enzyme activity, that is, CYP2C19*2 (G681A rs4244285, splicing defect) and CYP2C19*3 (G636A rs4986893, W212X, premature stop codon) have been reported^14,15^. The steady-state serum concentration of valproic acid is significantly dependent on the mutated alleles. However, there are large overlaps in these steady-state serum concentrations among the different genotype groups and considerable interindividual variations in the values within each genotype. In previous published studies, body weight, age, gender and comedications had significant influence on VPA distribution volume^16–18^. Although various genetic polymorphisms were associated with the increase or decrease of VPA serum concentration^6,12,19–24^, only two study identified the quantitative relationship between genetic polymorphisms *CYP2C19* and VPA distribution volume in epileptic patients^6,25^.

Therefore, we aimed to investigate the effects of various factors including CYP2C19 polymorphisms, age, gender, body mass index (BMI) and duration of schizophrenia on the steady-state serum concentrations of valproic acid in Chinese Han patients with schizophrenia, which might be useful for VPA dose adjustment in clinical practice.

## Materials and methods

### Participants

The subjects were 296 Chinese Han inpatients with schizophrenia (78 males and 218 females) who all fulfilled the criteria for schizophrenia. The inclusion criteria were as follows: (1) aged 18–60 years; (2) meeting the criteria of the International Classification of Diseases-Tenth Edition (ICD-10) for schizophrenia; (3) Han Chinese in origin; (4) normal liver and renal function; and (5) no substance abuse. The exclusion criteria included the following: (1) treatment-resistant schizophrenia; (2) pregnant or lactating women; and (3) with severe physical disease or brain organic disease, neurological disorder, delirium, or dementia. The mean ± SD of age and BMI value were 35.30 ± 12.40 years and 22.37 ± 4.14, respectively. 10 were smokers (≥ 10 cigarettes/day), whereas the remainders were nonsmokers. None was a heavy drinker. This study was approved by the Ethics Committee of the Hangzhou Seventh people’s hospital (2,016,018), and all the patients had given written informed consent to participate in this study. All methods were performed in accordance with the relevant guidelines and regulations.

### Drug treatment

All the subjects had received valproic acid for at least 2 weeks. It has been shown that plasma concentrations of valproic acid reach steady state by 2 weeks after repeated oral administration. The daily dose was fixed and was given twice a day. The patients were receiving no drugs except olanzapine (2.5–20 mg/day). Female patients did not receive oral contraceptives. Patients’ adherence was confirmed by the nursing staff or their families. Blood samples were taken at 8 a.m.

### Analytical methods

Serum concentrations of valproic acid were measured using the ultra-high performance liquid chromatography method with mass-spectrometric detection^26^ by Zhejiang BIOZON Medical Co., Ltd. The lowest limit of detection for valproic acid was 2.5 μg/ml using 0.4 ml of serum, and the interassay coefficient of variation was 7.98% at a concentration of 50 μg/ml. The steady-state serum concentrations were adjusted by the doses of valproic acid, and the concentration/dose (C/D) ratios were used in statistical analyses.

DNA was isolated from peripheral leucocytes using a nucleic acid extraction and purification kit (Kuangyuan, Suzhou, China). The *2 and *3 alleles of *CYP2C19* were identified by PCR analyses using Human *CYP2C19* genotyping kit (Kuangyuan, Suzhou, China).

### Statistical analyses

Demographic characteristics were compared by using Student's *t* test for continuous variables and *χ*^2^ test for categorical variables. The serum C/D ratios of valproic acid of patients were compared among different genotypic groups or different metabolic type groups using one way ANOVA analyses with Bonferroni post hoc tests. Multiple linear regression analyses were used to detect the association between serum C/D ratios of valproic acid and several factors, including the polymorphisms of CYP2C19, age, gender, BMI value, smoke and duration of schizophrenia. A *P* value of less than 0.05 was regarded as statistically significant. SPSS Statistics 23.0 for Windows was used for these statistical analyses. Post-hoc power analyses were calculated using G*Power software (version 3.1).

### Ethics approval and statement of consent to participate

The study was approved by the Ethics Committee of the Hangzhou Seventh People’s Hospital. The purpose and importance of the study were explained to each participant before they proceeded into actual activities. Informed consent was obtained from their guardians or relatives.

## Results

### Influence of *CYP2C19* genotype on serum C/D ratios of valproic acid

To explore the effects of *CYP2C19* genotype on interindividual variabilities in serum concentrations of valproic acid in Chinese Han schizophrenia, the CYP2C19*2 (G681A, splicing defect) and CYP2C19*3 (G636A, W212X, premature stop codon) were analyzed by real-time PCR. The characteristics of study population and the frequencies of *CYP2C19* genotype are shown in Table 1. There were no significant differences in the general characteristics among genotypes.

**Table 1:** Characteristics of study population.

	*1/*1	*2/*2	*3/*3	*1/*2	*1/*3	*2/*3	*F/χ*^2^	*p*
Male/female, n	31/87	9/21	0/0	28/90	7/12	3/8	1.707	0.790
Age, years	35.25 ± 11.87	33.17 ± 11.19	–	34.17 ± 13.51	32.79 ± 12.15	31.27 ± 9.49	0.472	0.756
BMI	22.62 ± 3.62	22.55 ± 3.52	–	22.30 ± 4.69	20.37 ± 3.10	23.43 ± 5.73	1.420	0.227
Duration of schizophrenia, month	124.31 ± 102.39	90.27 ± 74.80	–	110.64 ± 119.52	101.47 ± 90.33	125.27 ± 122.35	0.762	0.551

*1/*1: 681GG, 636GG; *1/*2: 681GA, 636GG; *1/*3: 681GG, 636GA; *2/*2: 681AA, 636GG; *2/*3: 681GA, 636GA; *3/*3: 681GG, 636AA.

**Figure 1:**
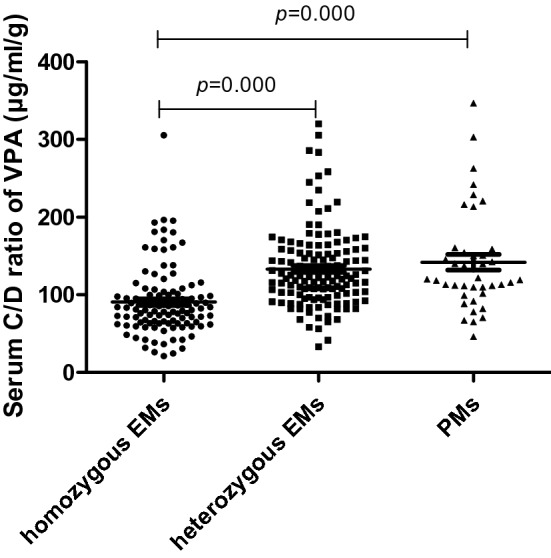
Differences in serum concentration/dose ratio of valproic acid in various metabolizer of CYP2C19 in Chinese schizophrenia patients. EMs: extensive metabolizers; PMs: poor metabolizers.

Furthermore, associations between *CYP2C19* SNPs and serum C/D ratios of valproic acid were observed. The number of patients with *CYP2C19* *1/*1, *1/*2, *1/*3, *2/*3, *2/*2 and *3/*3 genotype were 118, 118, 19, 11, 30 and 0, respectively (Table 2). Patients with CYP2C19 *1/*2 (*P* = 0.029) or CYP2C19 *2/*3 (*P* < 0.01) had significantly higher serum C/D ratios of valproic acid than those with CYP2C19 *1/*1 (Table 2). And, the mean concentration/dose ratios of valproic acid were significantly higher in patients with 1 (heterozygous extensive metabolizers, *P* < 0.01) or 2 (poor metabolizers, *P* < 0.01) mutated alleles for *CYP2C19* than in those without mutated alleles (Table 3 and Fig. 1). And the post hoc analysis revealed that the result has acceptable statistical (power (1 – β) = 0.8486 at type I level of 0.05) to support the observed significant associations for *CYP2C19* SNPs and serum C/D ratios of valproic acid.

**Table 2:** Steady-state serum C/D ratio of VPA between CYP2C19 genotypes.

Genotype	N	VPA C/D ratio (μg/ml/g)			
		mean	SD	SE	95% CI
681GG, 636GG	118	90.96	46.46	4.28	82.49–99.43
681AA, 636GG	30	130.76 a	54.35	9.92	110.46–151.05
681GG, 636AA	0	–	–	–	
681GA, 636GG	118	131.37 a	50.33	4.63	122.19–140.54
681GG, 636GA	19	132.42 a	32.42	7.44	116.80–148.05
681GA, 636GA	11	172.25 a	83.54	25.19	116.13–228.38

^a^*p* < 0.05 compared with group (681GG, 636GG).

**Table 3:** Steady-state serum C/D Ratio of VPA between CYP2C19 metabolic types.

metabolic types	N	VPA C/D ratio (μg/ml/g)			
		mean	SD	SE	95% CI
Homozygous EMs (*1/*1)	118	90.96	46.46	4.28	82.49–99.43
heterozygous EMs (*1/*2、*1/*3)	137	131.51 a	48.15	4.11	123.38–139.65
PMs (*2/*2、*2/*3、*3/*3)	41	141.89 a	65.06	10.16	121.35–162.43

^a^*p* < 0.01 compared with Homozygous Ems; power (1 – β) = 0.8486 at type I level of 0.05.

EMs: extensive metabolizers; PMs: poor metabolizers; *1/*1: 681GG, 636GG; *1/*2: 681GA, 636GG; *1/*3: 681GG, 636GA; *2/*2: 681AA, 636GG; *2/*3: 681GA, 636GA; *3/*3: 681GG, 636AA.

### Multiple regression analysis

Multiple regression analysis including *CYP2C19* polymorphisms, age, gender, BMI values, smoke and duration of schizophrenia revealed that the polymorphisms of *CYP2C19* (standardized beta = 12.480, *P* < 0.01) and the BMI values (standardized beta = − 1.518, *P* < 0.05) were correlated with C/D ratios of valproic acid (Table 4).

**Table 4:** Standardized partial correlation coefficients (Beta) between serum C/D ratio of VPA and various factors (n = 298, R^2^ = 0.170).

Variables	B	SE	Beta	t	*p*
(Constant)	99.380	21.735		4.572	0.000
CYP2C19 genotype	12.480	1.810	0.372	6.896	0.000
Gender	2.943	5.540	0.029	0.531	0.596
Age	0.606	0.332	0.138	1.826	0.069
BMI	− 1.518	0.706	− 0.116	− 2.151	0.032
Smoke	− 0.456	7.360	− 0.003	− 0.062	0.951
Duration of Schizophrenia	− 0.076	0.038	− 0.149	− 1.972	0.050

EMs: extensive metabolizers; PMs: poor metabolizers.

## Discussion

A number of reports have been published regarding the pharmacokinetics variability of antipsychotic. These data indicate substantial effects of select genotypes on the pharmacokinetics phenotype. As an important adjuvant drug in the treatment of schizophrenia, the blood concentration of VPA is also affected by genetic factors.

Although a number of reports have been published on the pharmacokinetic of valproic acid, mostly in epileptic patients, no study in schizophrenia patients. Therefore, we investigate the effects of CYP2C19 polymorphisms on the steady-state serum concentrations of valproic acid in Chinese Han patients with schizophrenia, which might be useful for VPA dose adjustment in clinical practice.

In this study, the mean serum C/D ratios of valproic acid increased in accordance with the number of the mutated alleles for *CYP2C19* in schizophrenia patients. It is the data that strongly implied that the mean serum C/D ratios of valproic acid increases in a gene dose-dependent manner. The mean concentration/dose ratios of valproic acid were significantly higher in patients with 1 or 2 mutated alleles for *CYP2C19* than in those without mutated alleles. Importantly, the post hoc analysis revealed that the result has acceptable statistical (power (1 – β) = 0.8486 at type I level of 0.05) to support the observed significant associations for *CYP2C19* SNPs and serum C/D ratios of valproic acid. The result was assistant with previous report in epileptic^3^, and it indicates that the influence of CYP2C19 polymorphism on serum concentration of VPA in schizophrenia is consistent with that of epilepsy patients. However, we just investigated the serum concentration of VPA in a very small part of schizophrenia patients treated with olanzapine and VPA. As for, that the effect of CYP2C19 polymorphism on serum concentration of VPA in schizophrenia patients treated with other antipsychotic drugs combined with VPA remains to be further observed.

In addition, multiple regression analyses showed that the serum C/D ratios of valproic acid were not only correlated with the number of mutated alleles for *CYP2C19* but also associated with the BMI values. One study showed that loss-of-function *CYP2C19* polymorphisms were associated with an increased BMI value^27^. This may explain the association between BMI values and the serum C/D ratios of valproic acid in our study. And some studies reported the effect of age on the metabolism of valproic acid^28,29^, showed that there was no statistically significant difference in the rate of VPA metabolism. Another study showed that serum valproic acid levels was significantly increased with younger age^30^. These results are inconsistent. A larger population sample size is needed to further verify the results.

This study has several limitations. Glucuronidation and mitochondrial beta-oxidation (approximately 70–90%) are the major VPA metabolic pathway in adults^31^. We did not investigate the contribution of uridine diphosphate glucuronosyltransferase polymorphisms which are the most confounding factors to interindividual variations in valproate pharmacokinetics^32,33^. We also did not investigate the contribution of CYP2C9 polymorphisms which are the important factors to valproate metabolism^10,12^. Second, the sample is too small to derive greater statistical power because of the limited number of patients eligible for the trial, but our research may provide guidance on VPA dosing in schizophrenia patients. Third, the dose of valproic acid was not unified. Data of clinical responses related with the observed differences in the steady-state serum concentrations of valproic acid were lacking.

## Conclusions

The findings of this study suggest that the genetic polymorphisms of CYP2C19 significantly affect the steady-state serum concentrations of valproic acid in Chinese Han population. The determination of the CYP2C19 genotypes may be useful for dosing adjustment in schizophrenia patients on valproic acid therapy.

## Data Availability

All data generated or analyzed during this study are included in this published article.

## References

[1] Christison GW, Kirch DG, Wyatt RJ (1991). When symptoms persist: Choosing among alternative somatic treatments for schizophrenia. Schizophr. Bull..

[2] Cipriani, A., Reid, K., Young, A. H., Macritchie, K. & Geddes, J. Valproic acid, valproate and divalproex in the maintenance treatment of bipolar disorder. *The Cochrane Database System. Rev.*10.1002/14651858.CD003196.pub2 (2013). 10.1002/14651858.CD003196.pub2 PMC6599863 24132760

[3] Citrome L, Levine J, Allingham B (2000). Changes in use of valproate and other mood stabilizers for patients with schizophrenia from 1994 to 1998. Psychiatr. Serv..

[4] Citrome L (2003). Schizophrenia and valproate. Psychopharmacol. Bull..

[5] Wang, Y., Xia, J., Helfer, B., & Li, C. Valproate for schizophrenia. *Cochrane Database System. Rev.* **11**, CD004028. 10.1002/14651858.CD004028.pub4 (2016). 10.1002/14651858.CD004028.pub4 PMC6734130 27884042

[6] Jiang D (2009). Effects of CYP2C19 and CYP2C9 genotypes on pharmacokinetic variability of valproic acid in Chinese epileptic patients: Nonlinear mixed-effect modeling. Eur. J. Clin. Pharmacol..

[7] Ghodke-Puranik Y (2013). Valproic acid pathway: Pharmacokinetics and pharmacodynamics. Pharmacogenet. Genomics.

[8] Zhong Z (2017). Analysis of CYP2C19 genetic polymorphism in a large ethnic Hakka population in southern China. Med Sci Monit.

[9] Wang C (2017). Association of CYP2C9, CYP2A6, ACSM2A, and CPT1A gene polymorphisms with adverse effects of valproic acid in Chinese patients with epilepsy. Epilepsy Res.

[10] Noushin AS (2010). Influence of CYP2C9 polymorphism on metabolism of valproate and its hepatotoxin metabolite in Iranian patients. Toxicol. Mech. Methods.

[11] Methaneethorn J (2018). A systematic review of population pharmacokinetics of valproic acid. Br. J. Clin. Pharmacol..

[12] Kiang TK (2006). Contribution of CYP2C9, CYP2A6, and CYP2B6 to valproic acid metabolism in hepatic microsomes from individuals with the CYP2C9*1/*1 genotype. Toxicol. Sci. J. Soc. Toxicol..

[13] Guo J (2020). Impact of gender, albumin, and CYP2C19 polymorphisms on valproic acid in Chinese patients: A population pharmacokinetic model. J. Int. Med. Res..

[14] Kurose K, Sugiyama E, Saito Y (2012). Population differences in major functional polymorphisms of pharmacokinetics/pharmacodynamics-related genes in Eastern Asians and Europeans: Implications in the clinical trials for novel drug development. Drug Metab. Pharmacokinet..

[15] Zanger UM, Schwab M (2013). Cytochrome P450 enzymes in drug metabolism: Regulation of gene expression, enzyme activities, and impact of genetic variation. Pharmacol. Ther..

[16] Ding J (2015). A population pharmacokinetic model of valproic acid in pediatric patients with epilepsy: A non-linear pharmacokinetic model based on protein-binding saturation. Clin. Pharmacokinet..

[17] Nakashima H (2015). Determination of the optimal concentration of valproic acid in patients with epilepsy: A population pharmacokinetic–pharmacodynamic analysis. PLoS ONE.

[18] Ogusu N (2014). Impact of the superoxide dismutase 2 Val16Ala polymorphism on the relationship between valproic acid exposure and elevation of gamma-glutamyltransferase in patients with epilepsy: A population pharmacokinetic-pharmacodynamic analysis. PLoS ONE.

[19] Budi T (2015). Clinical significance of CYP2C9-status guided valproic acid therapy in children. Epilepsia.

[20] Guo Y, Hu C, He X, Qiu F, Zhao L (2012). Effects of UGT1A6, UGT2B7, and CYP2C9 genotypes on plasma concentrations of valproic acid in Chinese children with epilepsy. Drug Metab. Pharmacokinet..

[21] Feng W (2016). Effects of UGT1A6 and GABRA1 on standardized valproic acid plasma concentrations and treatment effect in children with epilepsy in China. Ther. Drug Monit..

[22] Hung CC (2011). Association of genetic variants in six candidate genes with valproic acid therapy optimization. Pharmacogenomics.

[23] Mei S (2017). Genetic polymorphisms and valproic acid plasma concentration in children with epilepsy on valproic acid monotherapy. Seizure.

[24] Tan L (2010). The influence of cytochrome oxidase CYP2A6, CYP2B6, and CYP2C9 polymorphisms on the plasma concentrations of valproic acid in epileptic patients. Clin. Neurol. Neurosurg..

[25] Mei S (2018). Effect of CYP2C19, UGT1A8, and UGT2B7 on valproic acid clearance in children with epilepsy: A population pharmacokinetic model. Eur. J. Clin. Pharmacol..

[26] Chen, S. *et al.* Pseudotargeted metabolomics method and its application in serum biomarker discovery for hepatocellular carcinoma based on ultra high-performance liquid chromatography/triple quadrupole mass spectrometry. *Anal. Chem.*10.1021/ac4016787 (2013). 10.1021/ac4016787 23889541

[27] Noai M (2016). Cytochrome P450 2C19 polymorphisms and valproic acid-induced weight gain. Acta Neurol. Scand..

[28] Argikar UA, Remmel RP (2009). Effect of aging on glucuronidation of valproic acid in human liver microsomes and the role of UDP-glucuronosyltransferase UGT1A4, UGT1A8, and UGT1A10. Drug Metabol. Dispos. Biol. Fate Chem..

[29] Miyagi SJ, Collier AC (2011). The development of UDP-glucuronosyltransferases 1A1 and 1A6 in the pediatric liver. Drug Metabol. Dispos. Biol. Fate Chem..

[30] Ben Mahmoud, L. *et al.* Influence of age and co-medication on the steady-state pharmacokinetics of valproic acid in Tunisian patients with epilepsy. *Rev. Neurol.* **173**, 159–163. 10.1016/j.neurol.2017.02.004 (2017). 10.1016/j.neurol.2017.02.004 28320517

[31] Monostory K (2019). Relevance of CYP2C9 function in valproate therapy. Curr. Neuropharmacol..

[32] Munisamy M (2013). The effect of uridine diphosphate glucuronosyl transferase (UGT)1A6 genetic polymorphism on valproic acid pharmacokinetics in Indian patients with epilepsy: A pharmacogenetic approach. Mol. Diagn. Ther..

[33] Chu XM (2012). Influence of UDP-glucuronosyltransferase polymorphisms on valproic acid pharmacokinetics in Chinese epilepsy patients. Eur. J. Clin. Pharmacol..

